# Similarities in Quantitative Computed Tomography Imaging of the Lung in Severe Asthma with Persistent Airflow Limitation and Chronic Obstructive Pulmonary Disease

**DOI:** 10.3390/jcm10215058

**Published:** 2021-10-29

**Authors:** Andrzej Obojski, Mateusz Patyk, Urszula Zaleska-Dorobisz

**Affiliations:** 1 Department of Internal Diseases, Pneumonology and Allergology, Wroclaw Medical University, 50-369 Wrocław, Poland; andrzej.obojski@poczta.onet.eu; 2 Department of General and Pediatric Radiology, Wroclaw Medical University, 50-369 Wrocław, Poland; urszula.zaleska-dorobisz@umw.edu.pl

**Keywords:** asthma, chronic obstructive pulmonary disease, airway remodelling, emphysema, quantitative computed tomography

## Abstract

Background: Severe asthma with persistent airflow limitation (SA-PAL) and chronic obstructive pulmonary disease (COPD) are characterised by irreversible airflow limitation and the remodelling of the airways. The phenotypes of the diseases overlap and may cause diagnostic and therapeutic concerns. Methods: There were 10 patients with SA-PAL, 11 patients with COPD, and 10 healthy volunteers (HV) enrolled in this study. The patients were examined with a 128-multislice scanner at full inspiration. Measurements were taken from the third to ninth bronchial generations. Results: The thickness of the bronchial wall was greater in the SA-PAL than in the COPD group for most bronchial generations (*p* < 0.05). The mean lung density was the lowest in the SA-PAL group (−846 HU), followed by the COPD group (−836 HU), with no statistical difference between these two groups. The low-attenuation volume percentage (LAV% < −950 HU) was significantly higher in the SA-PAL group (15.8%) and COPD group (10.4%) compared with the HV group (7%) (*p* = 0.03). Conclusion: Severe asthma with persistent airflow limitation and COPD become similar with time within the functional and morphological dimensions. Emphysema qualities are present in COPD and in SA-PAL patients.

## 1. Introduction

Asthma and chronic obstructive pulmonary disease (COPD) are two major obstructive lung diseases constituting meaningful public health concerns [1]. COPD is the most common chronic lung disease in adulthood. The prevalence of COPD varies depending on the methodology used in epidemiological studies but remains very high. The estimated prevalence of COPD ranges from 7.6% [2] to 22.1% [3]. Asthma is the most common chronic lung disease in childhood. The incidence of asthma in the general population is as high as 11% [4]. In the last decades, a substantial increase in the morbidity, mortality, and economic burden of asthma and COPD has been observed worldwide [5].

Asthma and COPD are recognised to be chronic inflammatory airway diseases. However, the inflammatory process in both conditions differs meaningfully. Inflammation of airways leads to the narrowing of the bronchial tree and deterioration of the lung ventilatory function. Airflow obstruction in asthma is usually fully reversible, while in COPD, it is, by definition, irreversible. However, airflow obstruction in COPD is responsive to treatment [6,7]. Despite many similarities between these two diseases concerning the pathophysiology, clinical presentation, and therapy, both are distinct conditions and require accurate differential diagnosis. Currently, there are no available sufficiently specific and sensitive diagnostic tools to distinguish patients with severe asthma with persistent airflow limitation, COPD, and asthma/COPD overlap (ACO) [6,8]. In the absence of better diagnostic tools, the overlap is diagnosed in the majority of such subjects. In mild and moderate asthma, the airway narrowing is tidal and fully reversible, but in severe asthma, the airway obstruction often becomes permanent and less responsive to treatment. Under the circumstances, asthma with permanent airflow limitation may suggest a diagnosis of COPD. In the early phase of asthma, the narrowing of airways is mostly induced by bronchial smooth muscle contraction and slightly by a remodelling process, while in an advanced stage, fixed obstruction is mainly associated with airway wall remodelling [9]. Bronchial remodelling is a pathological feature of asthma and COPD that contributes to the symptoms and clinical presentation of the disease. It is believed that remodelling results from an inflammatory process triggered by the exposure to allergens, cigarette smoke, dust, and noxious chemicals. Bronchial remodelling is defined as a thickening of the bronchial wall due to the increase of smooth muscle mass, mucous gland hypertrophy, neoangiogenesis, and subepithelial membrane thickening. Fixed obstruction in the severe asthma clinically tends to mimic COPD. The overlap of the clinical presentation of severe asthma and COPD introduces challenges in clinical practice and remains a significant concern. Modern imaging techniques, including quantitative computed tomography, yield insight into the complexity of the airway structure and physiopathology of obstructive diseases [10,11,12].

It is generally accepted that asthma does not turn into emphysema. Although asthma and emphysema share some common properties, they are distinct diseases. However, the co-occurrence of asthma and emphysema is not a rare phenomenon [13]. Pulmonary three-dimensional, quantitative assessment may be helpful in the differentiation of composite kinds of emphysema and parenchymal changes. Thus, it may be potentially useful in asthma/COPD diagnosis and phenotyping [14,15]. The determination of the relationship between the airway remodelling, emphysema, and lung ventilatory function in asthma and COPD seems to be a promising advancement in clinical practice.

The primary aim of this study was to evaluate the intensity of the remodelling of the bronchi and the emphysema qualities of the lungs in patients with severe asthma with persistent airflow limitation (SA-PAL) COPD and healthy volunteers (HV) using quantitative computed tomography (QCT). The secondary aim was to establish the relationship between the remodelling and airflow limitation.

## 2. Materials and Methods

### 2.1. Patients Characteristics

We conducted a one-center observational study to compare the airway remodelling and parenchymal changes of the lungs in patients with SA-PAL, COPD, and HV. The protocol was reviewed and approved by the Bioethics Committee of the Wroclaw Medical University (No. KB—280/2014 and KB—92/2017) before the commencement of the study. All participants provided written informed consent before any study-related procedures.

The study was performed between 2016 and the end of 2018. Initially, we screened 40 participants. We collected data for 12 patients diagnosed with severe asthma, 13 patients diagnosed with moderate to severe COPD and 15 healthy subjects. The final analysis included 3 groups of patients who met the predefined inclusion criteria: 10 patients with SA-PAL, 11 patients with COPD, and 10 HV. All patients were recruited from the allergological outpatient clinic of the Department of Internal Diseases, Pneumonology and Allergology, Wroclaw Medical University.

The inclusion criteria for the SA-PAL group were as follows: severe asthma (fifth degree according to GINA 2015 guidelines), irreversible airway obstruction, age > 40, and clinically relevant perennial allergy to aeroallergen. The exclusion criteria for the SA-PAL group were as follows: smoking (current or in the past), diagnosis of COPD, diagnosis of any other lung disease, and diagnosis of other comorbidities with a potential impact on lung function. It should be noted that the diagnosis of asthma in every case was definite and had been made in childhood or adolescence. All SA-PAL subjects participated in the NHS treatment program with the omalizumab and formed a complete treatment group at the centre. Two patients were excluded from the final analysis: one participant was younger than 40 years old, while the other did not meet the criterion of perennial allergy.

The COPD subjects were recruited in a consecutive sampling at our centre. The inclusion criteria for the COPD group were as follows: GOLD 2–3 (moderate-severe airflow limitation), group D COPD, according to GOLD 2016 guidelines, age > 40, and current or past smoking with the history of ≥10 PY. The exclusion criteria for the COPD group were as follows: diagnosis of asthma, diagnosis of any other lung disease, and diagnosis of other comorbidities with a potential impact on lung function. In total, two patients were not included in the study: one due to the chest comorbidity (kyphoscoliosis), and one due to FEV1/FVC > LLN according to Global Lung Function Initiative (GLI2012).

Healthy volunteers were enrolled in nonprobability purposive sampling. In the healthy group, all subjects met the inclusion criteria, including no history of respiratory and heart disease, no history of any other clinically relevant condition, no current treatment, and no history of smoking and normal lung function according to GLI2012 reference values. Only patients older than 40 years were recruited to the control group. Five screened subjects were rejected: one due to FEV1/FVC below low limit of normal, two because of history of passive smoking, and two because of comorbidities in the past (more than one childhood pneumonia). The details of the baseline characteristics of the patients are provided in Table 1.

**Figure 1:**
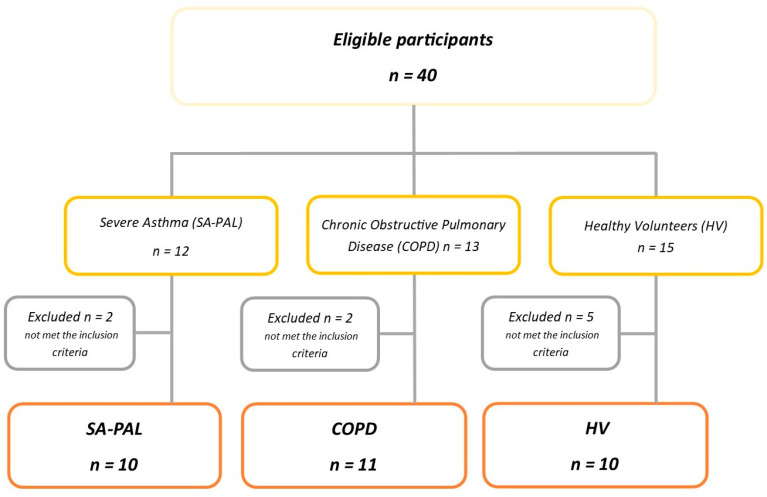
Patients’ flowchart.

The study procedures were performed in a stable phase of obstructive diseases, at least 3 months after the last exacerbation. Bronchodilators were withdrawn according to spirometry recommendations and drug characteristics to avoid impacts on the lung function [16]. The study procedures were performed in the morning and began with computed tomography (CT) followed by lung function tests (LFT). Figure 1 presents the detailed flowchart of the examined participants.

### 2.2. Computed Tomography Scanning

CT was carried out in the supine position and the craniocaudal direction at full inspiration and breath-holds using a 128-multislice CT scanner (SOMATOM Definition AS+, Siemens Healthcare, Erlangen, Germany) with a non-contrast chest scanning protocol. The Syngo.Pulmo3D software (Siemens Healthcare, Erlangen, Germany) was used to obtain the quantitative reconstructions and parameters of the lung. The airway measurements were performed from the third (segmental) to the ninth generation of the subsegmental bronchi of the right lung (RB10). All measurements were carried out by one observer blinded to the clinical data in cross-sectional scans, perpendicular to the long axis, with the multi-planar image reconstructions (MPR) in the middle part of bronchi generation (Syngo.Pulmo3D, Siemens Healthcare, Erlangen, Germany). The measurements of the mean wall thickness (WT), wall area (WA), wall area percentage (WA%), and lumen area (LA) were performed.

Lung densitometry post-processing software was applied to assess the structure of the parenchyma of the lung. The mean lung density (MLD) was measured to identify the areas of tissue loss or condensation and was presented in Hounsfield Units (HU) [17]. The −950 HU threshold for the inspiratory CT scans was considered as the limit value and representative of lung emphysema [14].

The areas of the reduced tissue density were detected at a low-attenuation volume (LAV) and expressed as the percentage of the lung volume LAV%. A ratio over 10% was considered as emphysema [18,19]. Immediately after CT, all subjects performed a lung function test (LFT) using a Jaeger MasterScope device according to the ATS and GINA guidelines [16]. The following parameters were measured: forced expiratory volume in 1 s (FEV1) and forced vital capacity (FVC). Then, the FEV1/FVC% was calculated.

The full methodology of CT scanning, lung function testing, and statistical analysis were described in the associated publication: Patyk M. et al. (2020) [20].

### 2.3. Statistical Analysis

The GraphPad Prism v. 7.0.0 (GraphPad Software, San Diego, CA, USA) was used to compute the statistical analysis. Because of the small number of examined groups, the Mann–Whitney test was used to calculate the statistical differences between the parameters of the two groups. To compare three groups, the Kruskal–Wallis test was used. The *p*-value < 0.05 was considered significant. The Spearman-r test was applied to calculate the correlation coefficients between the factors.

## 3. Results

The airway segmentation from third to seventh generations of the bronchi was possible in the majority of the examined subjects, i.e., in 93.5% (*n* = 29). The eighth generation was obtained in 87.1% (*n* = 27) of the subjects, and the ninth was obtained in 58.1% (*n* = 18) of the subjects.

**Figure 2:**
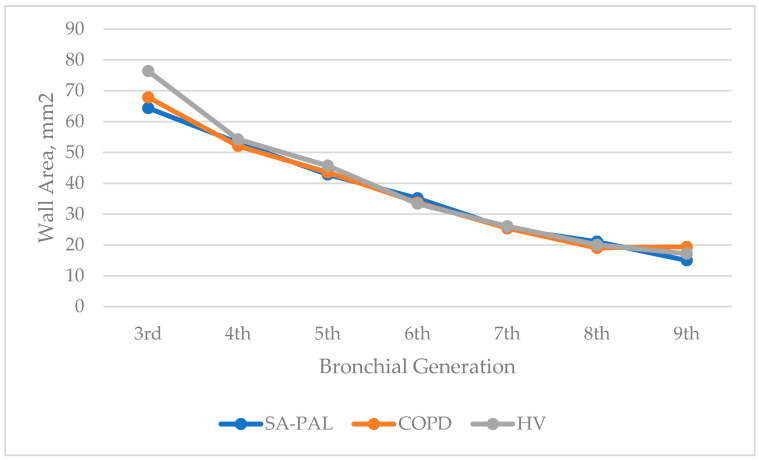
Wall area (WA) of the bronchial tree generations in patients in the SA-PAL, COPD, and HV groups.

**Figure 3:**
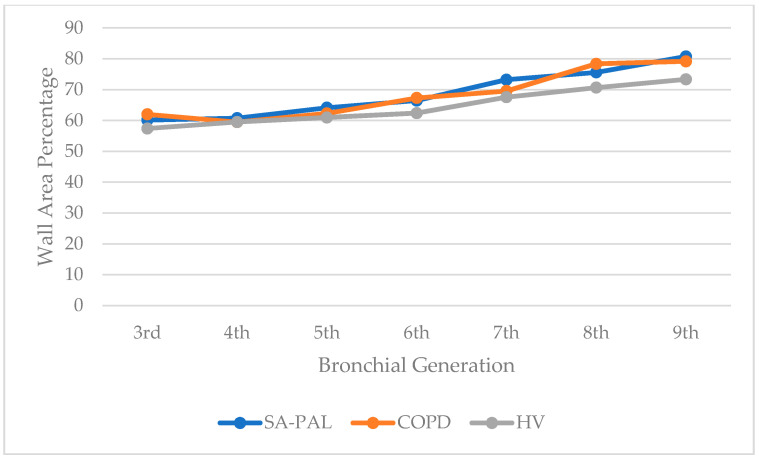
Wall area percentage (WA%) of the bronchial tree generations in patients in the SA-PAL, COPD, and HV groups.

**Figure 4:**
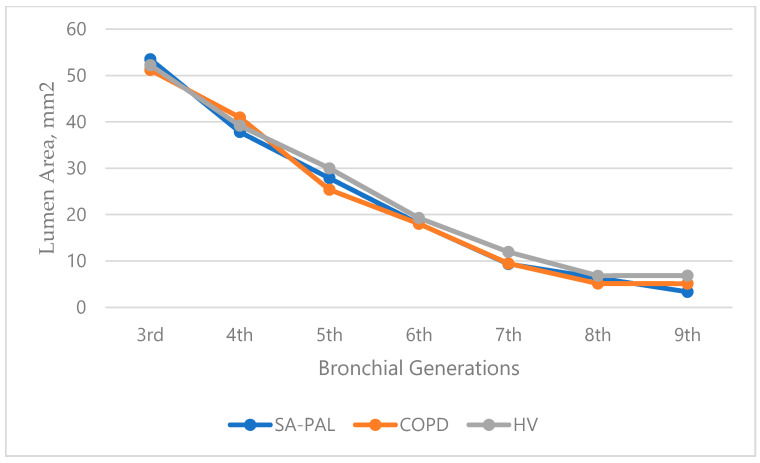
Lumen area (LA) of the bronchial tree generations in patients in the SA-PAL, COPD, and HV groups.

There was a significant increase in the WT in the SA-PAL and COPD patients compared with the HV subjects. The airway thickness was consistently and statistically significantly increased from the third to ninth bronchi generations in the asthma group, and from the fifth to ninth bronchi generations in the COPD group (*p* < 0.05). Interestingly, the airway thickness was also increased in numerical values in the asthma group compared with the COPD group throughout all examined bronchi generations and reached a statistical significance in third and fourth, and seventh and eighth bronchi generations. The details of the WT are shown in Table 2. The WA% was increased in the SA-PAL and COPD groups compared with the healthy subjects. However, it did not reach statistical significance (*p* > 0.05). There was no statistically significant difference regarding the WA, WA%, and LA between the obstructive groups and the healthy subjects. However, a noticeable decrease in the figures in the LA, as well as an increase in the WA% in the SA-PAL group, were noticed. The changes were the most noticeable in the distant bronchi from the fifth and sixth generations to the ninth generation. The WA of all bronchial generations were similar in all groups (*p* > 0.05). The values of the WA, WA%, and LA are presented in Figure 2, Figure 3 and Figure 4.

The data analysis showed no correlation between all airway measurements and lung function as measured by FEV1% and FVC%. No statistically significant correlation was demonstrated for the WT, WA%, and LA in all studied groups. The parenchyma parameters demonstrated the lowest MLD in the asthma and COPD groups. The difference between the SA-PAL and HV group was statistically significant (*p* < 0.05), while the difference between the COPD and HV group was close to the significance threshold (*p* = 0.085). Interestingly, there was no difference between the SA-PAL and COPD group. The LAV% was the highest in the SA-PAL group, followed by the COPD and HV group. The differences between all groups were statistically significant, with significantly higher figures in the obstructive groups. The details of the parenchyma characteristics are provided in Table 3.

## 4. Discussion

Asthma and COPD are the two most common obstructive lung diseases. Despite the clear correspondence between the diseases, both remain different nosological and clinical entities. They are characterised by different aetiologies, pathophysiologies, courses, and clinics. Nevertheless, due to the bilateral similarities of clinical presentation, the differentiation of the severe stages of asthma and COPD can be problematic in clinical practice [8,21,22,23]. The primary example of the difficulty is severe asthma with fixed obstruction that mimics COPD [6]. A clinical overlap may lead to challenges in choosing the optimal treatment. In the presented study, we compared patients with severe asthma with documented persistent airflow limitation, COPD patients, and healthy volunteers. The subjects did not differ significantly regarding height, BMI, and sex (*p* > 0.05). The COPD patients were older than the asthma patients and healthy volunteers (64.0 vs. 53.5 and 52.0 years old, respectively; *p* < 0.05), which was related to the late onset of COPD. The patients with COPD also presented more numerically severe airway obstruction compared with the patients with asthma (median FEV1% 43.50% vs. 56.25%, respectively) but the difference did not reach statistical significance (*p* > 0.05). Although there was no difference in the statistical evaluation, it may be of clinical significance. The vital part of the study was the reliability of the diagnosis of the enrolled patients. In each case, the diagnosis was of the highest diagnostic confidence and did not raise any doubts. In the asthma group, all patients had their diseases diagnosed at an early stage, had a clinically significant airborne allergy, and were never smokers. In the COPD group, the diagnosis was made in adults, over 40 years, all patients were current or ex-smokers, and no one presented symptoms of asthma or allergic rhinitis. The study procedures were based on modern imaging techniques and post-processing radiology.

Despite raising questions concerning the management of severe asthma, COPD, and ACO, there is still limited knowledge and unmet needs in the research on imaging biomarkers in obstructive diseases. Post-processing techniques and post-processing data analysis, i.e., quantitative computed tomography, are the modern extensions of the conventional high-resolution technique. The quantitative computed tomography was shown to yield accurate and reliable data of the airways and lung parenchyma [5,24]. In most of the performed studies, bronchial reconstruction has mainly concerned the proximal airways, up to the fifth and sixth generations [11,12,25,26]. The lower, more distal bronchi were not accessible due to the older types of CT scanners, lower imaging resolution, significant artefacts, and software limitations [27]. In our study, most of the subjects had the reconstruction of the bronchi performed up to the ninth generation. In a large single-centre study, Grydeland et al. researched the remodelling of the airways and lungs in COPD patients and non-COPD current or former smokers. Asthmatic patients were not excluded. They used a conventional high-resolution CT (HRCT) scanning protocol to perform the measurements of the lung, including the percentage of low-attenuation area <−950 HU (%LAA) and airway wall thickness. The measurements were performed in the large bronchi with the internal diameter larger than 6 mm. They found that the increase of the %LAA and WT was related significantly to the reduced DLCO in both the COPD and non-COPD subjects [28]. In 2018, Li et al. presented retrospective imaging data of the fifth to the seventh bronchi generations in COPD patients. The study did not include a healthy group for comparison. The authors documented a significant and consistent difference in the ID, WT, LA, and WA% between the male and female COPD patients [29]. The WA% was larger, and the ID, WT, and LA were smaller in the women than the men in all groups, regardless of the smoking status (smoking, smoking cessation, non-smoking). Interestingly, the FEV1% was significantly lower in the women than the men, but only in the smoking and smoking cessation groups. In the non-smoking group, the FEV1% was significantly higher in the women than in the men. In the presented study, we demonstrated significantly increased bronchial wall thickness in the asthma and COPD patients compared with the healthy individuals. It is assumed that the wall thickness of the bronchi translates into the remodelling phenomenon. The wall thickness was significantly greater in all bronchi generations (WT3–WT9) in the asthma group and only more distal airways in the COPD group (WT5–WT9). This suggests a different model for the development of the remodelling in asthma and COPD. This finding is consistent with the results of previous studies in both asthma and COPD [30]. It is worth noting that the wall thickness was more expressed in the severe asthma patients compared with the COPD patients throughout the bronchial tree (WT3–WT4 and WT7–WT8). This phenomenon occurred despite the less advanced airflow limitation in the SA-PAL group compared to the COPD group (FEV1% 56.25 vs. 43.50%, respectively (*p* = 0.19)). We also observed no correlation between the airway measurements and lung function as measured by the FEV1% and FVC%. The findings suggest that the remodelling plays a pivotal role in obstructive diseases, but it is not the only mechanism responsible for airflow limitation. The observation requires further research on larger groups of patients. The difference in the intensity and distribution of remodelling also suggests the different pathophysiology of asthma and COPD and advocates their distinct nature. The percentage of the wall area (WA%) was increased in both obstructive groups compared with the healthy individuals. However, it did not reach statistical significance. We hypothesise that the lack of significance comes from the small number of enrolled subjects. We observed similar results for the inner diameter of the airways. The ID was smaller in both obstructive groups compared with the healthy group. However, it does not reach the statistical significance. The trend toward the smaller inner diameter was more expressed in the more distal bronchi. The remaining parameters of the respiratory tract, particularly the lumen area (LA), did not differ significantly between the groups. Our results are consistent to some extent with the observations performed by Hartley et al. in 2016 and Górska et al. in 2009. Hartley and colleagues also confirmed the presence of the airway remodelling in asthmatic and COPD patients. It is possible that larger groups of enrolled subjects allowed the researchers to show significant differences not only in the WT, but also in the other parameters of the remodelling, including the WA% [9]. The comparison of the patients with mild to moderate asthma and COPD performed by Górska et al. also demonstrated an increased thickness of the bronchial wall in both groups but without statistical significance [31].

Emphysema is a histological diagnosis and is defined as permanent damage of the alveolar walls and small airways distal to the terminal bronchioles, without obvious fibrosis. Emphysema is usually accompanied by airflow limitation and air trapping identified on CT scans as areas of lower attenuation and lower mean lung density [32,33]. Emphysematous changes on CT are highly reproducible and are consistent with histologically proven emphysema [34]. For the safety of the enrolled patients, we decided to perform only one CT scan on the inspiration. Therefore, the air trapping was not assessed. In the study, we adopted a commonly accepted threshold for emphysema of −950 HU for the inspiratory CT scans [14]. Emphysema was quantified by the percentage of lung voxels with attenuation values below −950 HU (LAV%). The cut-off value for LAV% at which emphysema is diagnosed is still under discussion and ranges between 6% to 15% [33,35]. We adopted a rate above 10% [18,19].

The densitometry of the lung parenchyma revealed a significant lung hyperinflation in both obstructive disease groups compared with the healthy subjects. The mean lung density in the asthma group (−846 HU) was the lowest and showed a statistically significant difference compared with the healthy group (−808 HU; *p* = 0.02). The difference between the asthma and COPD groups was minimal and did not reach statistical significance (−846 HU and −836, respectively; *p* = 0.67). Concerning the low-attenuation volume percentage value (LAV%), the difference between the asthma and COPD groups and the healthy group was even more expressed and statistically significant. It is interesting that the changes were more expressed in the asthma group than in the COPD group. However, in both groups, the LAV% <−950 HU exceeded 10% (15.8% and 10.4%, respectively; *p* = 0.02). The less extensive LAV% in the COPD group may be related to the heterogeneity of COPD, uneven distribution of chronic bronchitis, and emphysema in individual patients, as well as the small number of subjects.

Regardless of the histological nature of an emphysema definition, it seems reasonable to term areas of low attenuation below −950 HU as emphysema [14,35,36,37]. In light of the above assumption, our results are contrary to the statement that asthma does not turn into emphysema. SA-PAL and COPD may show a similar emphysematous pattern in lung imaging. This observation is not isolated. In a series of articles, Gelb et al. demonstrated the presence of centrilobular emphysema in patients with asthma and non-reversible airflow limitation [38]. The authors suggested that some patients with no history of smoking and with a normal α-1-antitripsin level can still develop the emphysema due to a chronic inflammatory reaction that triggers proteolytic destruction of the lung tissue [39]. In recent studies, Gelb et al. reported five cases of autopsy-proven, diffuse centrilobular emphysema in patients with chronic asthma [39,40]. Different results were obtained in the study by Hartley et al. [9]. Emphysema was shown to be present only in the COPD group. The discrepancy in the results may arise from the adopted definition of emphysema in the Hartley study (15% as the cut-off point for voxels below −950 HU) and the different populations. In the Hartley study, the asthma patients’ population presented less severe airflow limitation (pre-BD FEV% 78.2%) [9]. Based on our results, we hypothesise that the emphysema in severe asthma with a non-reversible airflow obstruction is a possible clinical presentation. This view brings a new debating point in the discussion about asthma and COPD overlap. The study did not address the issue of how to differentiate between the overlapping clinical presentations of SA-PAL and COPD. The answer to this question requires further research. A potential research direction is the evaluation of clusters of obstructive disease traits, including the morphology of distal generations of the airways, the pattern of severity, and distribution of emphysema, inflammatory biomarkers, and comprehensive pulmonary function tests.

The conclusions from our study present some limitations. The most recognisable is the small size of the examined groups. It is, in part, counterpoised by the very precise enrolment of asthma and COPD patients and the appropriate matching of all groups. The presented study may be a signal for the discussion and inspiration for further research in this field. The minor disadvantage is the resignation from the measurement of air trapping. The decision was forced by the choice of scanning protocol and safety reasons. The last limitation is an incomplete lung function testing, which was limited to the spirometry only. The use of other lung function tests, i.e., DLCO and plethysmography, are projected in future studies.

## 5. Conclusions

Severe asthma with persistent airflow limitation and COPD may show some morphological similarities over time. The remodelling of the bronchi was more expressed in the SA-PAL group than in the COPD group. Wall thickening was diffused along the bronchial tree in severe asthmatics (WT3–WT9), whereas it involved bronchi between the fifth to ninth generations in the COPD group (WT5–WT9). Emphysema is a phenomenon reserved that is not only to the COPD but can also occur in SA–PAL. The differences in the mechanisms of the remodelling and lung parenchymal changes in asthma and COPD require further research. The presented results raise a question about the relationship between COPD, severe asthma, and ACO. A better understanding of pathophysiology may contribute to a better phenotyping of obstructive diseases and possibly to more precise and phenotype-driven treatments.

## Figures and Tables

**Table 1:** Patients’ characteristics.

	SA-PAL (*n* = 10)	COPD (*n* = 11)	HV (*n* = 10)	SA-PAL vs. HV ^1^	SA-PAL vs. COPD ^1^	COPD vs. HV ^1^	Kruskal-Wallis Test
	M (25Q ÷ 75Q)	M (25Q ÷ 75Q)	M (25Q ÷ 75Q)	*p*-Value	*p*-Value	*p*-Value	*p*-Value
Age	53.5 (45.5 ÷ 53.5)	64.0 (60.0 ÷ 68.0)	52.0 (50.0 ÷ 58.3)	0.52	0.0002	0.0003	0.0004
Height, cm	167 (161 ÷ 177)	170 (158 ÷ 180)	173 (169 ÷ 178)	0.28	0.88	0.31	0.45
BMI	28.99 (23.29 ÷ 30.46)	29.01 (24.15 ÷ 33.9)	27.5 (23.7 ÷ 32.4)	0.91	0.61	0.71	0.83
FEV1, L	1.67 (1.23 ÷ 1.94)	1.12 (0.78 ÷ 1.58)	3.44 (2.84 ÷ 4.08)	<0.0001	0.04	<0.0001	<0.0001
FEV1%	56.25 (43.75 ÷ 65.63)	43.5 (31.25 ÷ 63.25)	110.1 (98.08 ÷ 127.50)	<0.0001	0.19	<0.0001	<0.0001
FVC, L	3 (2.65 ÷ 3.57)	2.57 (1.94 ÷ 3.47)	4.24 (3.62 ÷ 5.68)	0.003	0.20	0.0005	0.001
FVC%	91.25 (77.23 ÷ 110.50)	84.5 (74.75 ÷ 89.00)	119.4 (106.10 ÷ 131.90)	0.01	0.31	0.0001	0.001
FEV1/FVC%	51.88 (44.43 ÷ 62.30)	41 (35.25 ÷ 53.00)	76.68 (71.88 ÷ 83.36)	0.0001	0.06	<0.0001	<0.0001

SA-PAL—Severe Asthma with persistent airflow limitation, COPD—Chronic Obstructive Pulmonary Disease, HV—Healthy Volunteers, M—median; 25Q—25th quartile; 75Q—75th Quartile, ^1^ Mann–Whitney *t*-test.

**Table 2:** Wall thickness.

		SA-PAL		COPD		HV	SA-PAL vs. HV ^1^	SA-PAL vs. COPD ^1^	COPD vs. HV ^1^	Kruskal–Wallis Test
	*n*	M (25Q ÷ 75Q)	*n*	M (25Q ÷ 75Q)	*n*	M (25Q ÷ 75Q)	*p*-Value	*p*-Value	*p*-Value	*p*-Value
WT3, mm	10	2.19 (1.96 ÷ 2.59)	11	1.68 (1.51 ÷ 2.03)	10	1.76 (1.64 ÷ 2.02)	0.005	0.006	0.88	0.008
WT4, mm	10	2.03 (1.87 ÷ 2.32)	11	1.84 (1.39 ÷ 2.00)	10	1.62 (1.53 ÷ 1.82)	0.009	0.049	0.48	0.02
WT5, mm	10	1.84 (1.66 ÷ 2.09)	11	1.86 (1.56 ÷ 1.91)	10	1.57 (1.29 ÷ 1.75)	0.01	0.37	0.04	0.03
WT6, mm	10	1.63 (1.57 ÷ 1.83)	11	1.63 (1.42 ÷ 1.73)	10	1.4 (1.24 ÷ 1.53)	0.0002	0.39	0.003	0.001
WT7, mm	8	1.67 (1.58 ÷ 1.71)	11	1.36 (1.33 ÷ 1.62)	10	1.27 (1.14 ÷ 1.36)	<0.0001	0.03	0.01	0.0005
WT8, mm	8	1.57 (1.40 ÷ 1.67)	9	1.42 (1.22 ÷ 1.56)	10	1.21 (1.08 ÷ 1.31)	0.0003	0.02	0.04	0.009
WT9, mm	5	1.36 (1.27 ÷ 1.52)	6	1.32 (1.29 ÷ 1.40)	7	1.2 (1.04 ÷ 1.24)	0.003	0.63	0.006	0.007

SA-PAL—Severe Asthma with persistent airflow limitation, COPD—Chronic Obstructive Pulmonary Disease, HV—Healthy Volunteers, M—median; 25Q—25th quartile; 75Q—75th Quartile, ^1^ Mann–Whitney *t*-test.

**Table 3:** Lung parenchyma parameters in the SA-PAL, COPD, and HV groups.

		SA-PAL		COPD		HV	SA-PAL vs. HV ^1^	SA-PAL vs. COPD ^1^	COPD vs. HV ^1^	Kruskal–Wallis Test
	*n*	M (25Q ÷ 75Q)	*n*	M (25Q ÷ 75Q)	*n*	M (25Q ÷ 75Q)	*p*-Value	*p*-Value	*p*-Value	*p*-Value
Lung Volume	10	4826 (4567 ÷ 6191)	11	5908 (4507 ÷ 7089)	10	4577 (4258 ÷ 6970)	0.67	0.56	0.54	0.69
MLD, HU	10	−846 (−862 ÷ −815)	11	−836 (−860 ÷ −806)	10	−808 (−830 ÷ −791)	0.02	0.67	0.085	0.06
SD, HU	10	170 (164 ÷ 175)	11	165 (154 ÷ 183)	10	175 (167 ÷ 183)	0.18	0.67	0.17	0.27
LAV, %	10	15.8 (7.58 ÷ 19.50)	11	10.4 (5.50 ÷ 19.50)	10	7 (4.00 ÷ 8.60)	0.03	0.02	0.03	0.05

SA-PAL—Severe Asthma with persistent airflow limitation, COPD—Chronic Obstructive Pulmonary Disease, HV—Healthy Volunteers, M—median; 25Q—25th quartile; 75Q—75th Quartile, ^1^ Mann–Whitney *t*-test.

## Data Availability

All relevant data are within the paper.
